# VEGF-A mRNA processing, stability and translation: a paradigm for intricate regulation of gene expression at the post-transcriptional level

**DOI:** 10.1093/nar/gkt539

**Published:** 2013-07-12

**Authors:** Tania Arcondéguy, Eric Lacazette, Stefania Millevoi, Hervé Prats, Christian Touriol

**Affiliations:** ^1^Inserm UMR1037, Centre de Recherches en Cancérologie de Toulouse, CHU Rangueil, BP84225, 31432 Toulouse Cedex 4, France and ^2^Université Toulouse III Paul-Sabatier, 118 Route de Narbonne, 31400 Toulouse, France

## Abstract

Vascular Endothelial Growth Factor A (VEGF-A) is a potent secreted mitogen crucial for physiological and pathological angiogenesis. Post-transcriptional regulation of VEGF-A occurs at multiple levels. Firstly, alternative splicing gives rise to different transcript variants encoding diverse isoforms that exhibit distinct biological properties with regard to receptor binding and extra-cellular localization. Secondly, VEGF-A mRNA stability is regulated by effectors such as hypoxia or growth factors through the binding of stabilizing and destabilizing proteins at AU-rich elements located in the 3′-untranslated region. Thirdly, translation of VEGF-A mRNA is a controlled process involving alternative initiation codons, internal ribosome entry sites (IRESs), an upstream open reading frame (uORF), miRNA targeting and a riboswitch in the 3′ untranslated region. These different levels of regulation cooperate for the crucial fine-tuning of the expression of VEGF-A variants. This review will be focused on our current knowledge of the complex post-transcriptional regulatory switches that modulate the cellular VEGF-A level, a paradigmatic model of post-transcriptional regulation.

## INTRODUCTION

Gene expression was previously dominated by two main concepts. The first includes the « one gene, one protein » rule implying that one eukaryotic transcriptional unit encoded for a single open reading frame, resulting in a single gene product. The second concept largely documented, considers that regulation of gene expression occurs essentially at the transcriptional level. Today, we have moved on from these basic ideas. Indeed, the human genome is composed of <25 000 genes, far fewer than had been predicted before it was sequenced. It is a relatively low number of genes compared with less complex organisms such as *Drosophila melanogaster* (13 601 genes) or *Caenorhabditis elegans* (18 424 genes). This observation highlights the importance of mechanisms leading to protein diversity by alternative modes of gene expression, enabling an organism to increase its level of complexity. In fact, human cells have the ability to express ∼100 000 proteins using in part all facets of transcriptional and post-transcriptional regulations. These regulations include the use of alternative promoters, splicing sites, polyadenylation sites or translation initiation codons.

Among countless genes whose expression is tightly controlled at different levels, the Vascular Endothelial Growth Factor A (VEGF-A) gene is regulated at all conceivable stages of gene expression, and consequently represents a paradigm for gene regulation.

Blood vessel formation, an extremely tightly regulated process known as angiogenesis, is essential for proper organ growth and repair. Imbalances in the regulation of this process contribute to inflammatory, ischemic, immune or malignant disorders. The VEGF-A is a growth and survival factor for endothelial cells, playing an essential role in physiological and pathological angiogenic processes throughout embryonic development and during adulthood (1,2). VEGF-A affects both the development of new blood vessels (angiogenesis) and the survival of endothelial cells (vascular maintenance) by binding to the two tyrosine kinase receptors VEGFR1 and VEGFR2 (3,4).

In the quiescent vasculature of adult organs, basal levels of VEGF-A protect endothelial cells from apoptosis. However, the VEGF-A level increases in a number of physiological situations, such as oestrus, wound repair and adaptation to hypoxia or several pathological states such as proliferative retinopathies, arthritis, psoriasis and cancer (5–7). In addition, VEGF-A acts as the key mediator of tumour angiogenesis by stimulating the growth of new blood vessels from nearby capillaries, allowing tumour cells to acquire oxygen and nutrients and to metastasize.

Deletion of a single VEGF-A allele (8,9) results in embryonic lethality due to incorrect vascularization. The finding that a modest over-expression (10) results in a defective vascularization and early embryonic death emphasizes the role of VEGF-A in developmental angiogenesis.

Tissue-induced over-expression of VEGF-A in adult mice affects angiogenesis and vascular hyperpermeability (11), the formation of angiomas and organ development (12) or causes severe proliferative retinopathy and retinal detachment (13). Moreover, conditional transgenic knock-out models of VEGF-A have impaired vascular functions (14,15). Finally, conditional gain and loss of function of VEGF-A in the erythroid lineage demonstrated that alteration of VEGF-A levels during development significantly modulated erythropoiesis in mice embryos (16). To conclude, transgenic mice experiments have clearly demonstrated that VEGF-A expression is tightly regulated during development or in the adult: a slight variation of its normal level can induce deleterious phenotypes.

Consequently, the need for finely tuned VEGF-A expression is highlighted by a complex regulation at multiple levels, including transcriptional regulation, mRNA stabilization, alternative splicing and translational regulation as well as differential cellular/extracellular localization of various isoforms.

Recent research has produced significant improvements in our understanding of post-transcriptional regulation of VEGF-A, including the identification of novel trans-acting protein factors regulating mRNA stability and cis-elements such as two internal ribosome entry sites (IRES), two alternative initiation codons within the 5′ untranslated region (5′UTR) and multiple functional miRNA target sites within the 3′ untranslated region (3′UTR). In addition, it has been reported that the VEGF-A 5′UTR possesses anti-apoptotic and tumour-promoting activities independently from the VEGF-A open reading frame (ORF). The purpose of this review is to summarize the current knowledge on VEGF-A expression with particular emphasis on the multiple levels of post-transcriptional regulation.

## VEGF-A GENE ORGANIZATION AND TRANSCRIPTIONAL REGULATION

The human *VEGFA* gene, located on chromosome 6 at 6p21.1 (17), is organized into eight exons and seven introns and encompasses approximately 14 kb (18).

The *VEGFA* promoter sequence spans 1.2 kbp in the mouse and the rat gene (19,20) whereas a region of 2.36 kbp has been demonstrated to be critical in the human gene (21). The promoter lacks a consensus TATA box, but contains several consensus-binding sites for transcriptional regulators, including AP1, AP2 and Sp1, regulators dependant on growth factors, cytokines, hormones, tumour suppressor genes and oncogenes [for a review (22)].

Among stimuli responsible for the transcriptional up-regulation of the *VEGFA* gene, hypoxia has been of particular interest because of its role in cancer progression. It is now well established that the HIF (Hypoxia Inducible Factor) transcriptional activators are key mediators of hypoxic responses. Forsythe and collaborators initially identified a functional hypoxia response element (HRE) within the human 5′ flanking region element, which is the target of HIF-1 (23) and also HIF-2 (24).

**Figure 1.**
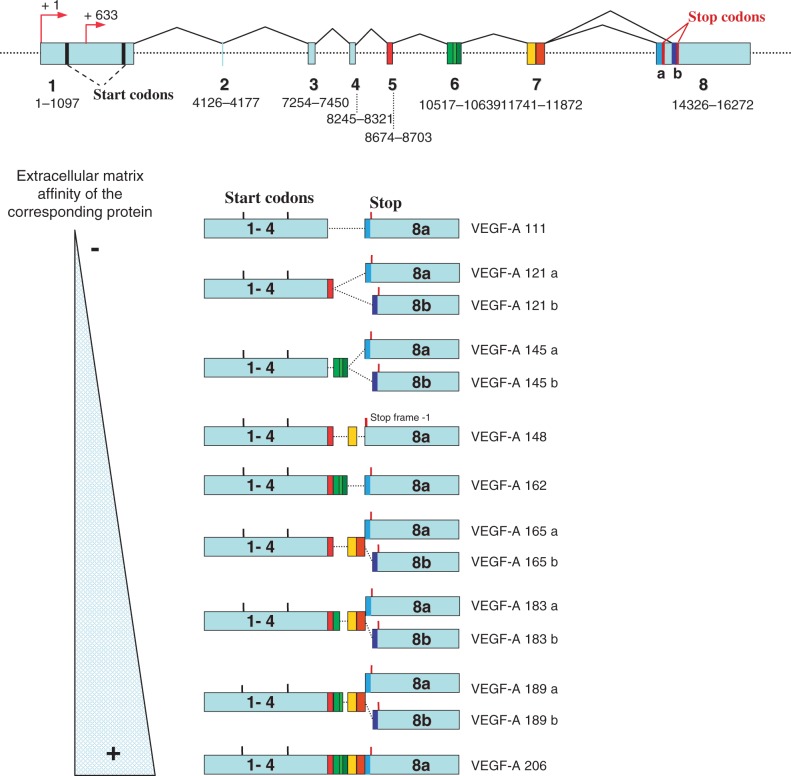
Human *VEGFA* gene structure and exon composition of the isoforms generated by alternative splicing. *VEGFA* gene spans 16 272 bp of chromosome 6p12 and consists of eight exons and seven introns. The two major transcription start sites, the two translation start sites (AUG and CUG) in the first exon and the two alternative stop codons in exon 8 are indicated. All currently described isoforms (named according to the amino acid number of the human synthesized protein) contain exons 1–5 and two different exons 8. The selection of the terminal exon splice site results in two isoform families, the pro-angiogenic VEGF-Axxx family and the antiangiogenic VEGF-Axxxb family. Exons 6 and 7 encode heparin-binding domains, responsible for the diffusibility and extra cellular matrix affinity of the alternative spliced isoforms.

An alternative promoter located within the human VEGF-A 5′UTR was described in 1998 by Akiri *et al.* (25) This transcription start site is located 633 nucleotides downstream of the main starting site (Figure 1). Insensitivity of this alternative promoter to hypoxia indicates no cross-regulation with the main promoter region and the potential of regulation under distinct conditions (25). Thus, these two transcripts may have different spatiotemporal expression patterns.

Nevertheless, transcriptional regulation accounts for only a fraction of the *VEGFA* gene expression compared with post-transcriptional mechanisms that play a crucial role in the fine-tuning of this factor.

## VEGF-A POST-TRANSCRIPTIONAL REGULATION

### Alternative splicing

During the splicing, alternative exons can either be retained in the mature messenger or targeted for removal in different combinations to create a diverse array of mRNAs from a single pre-mRNA. In addition, alternative splicing within non-coding regions of the mRNA can result in deletion of regulatory elements such as translation enhancers or RNA stability domains that may significantly affect the level of protein expression. Alternative splice events altering the protein-coding region of the mRNA will generate proteins which differ in their amino acid sequence and therefore in their activities.

As a typical secreted protein, VEGF-A has an N-terminal signal peptide consisting of 26 hydrophobic amino acids encoded by exon 1. VEGF-A also exists as multiple isoforms resulting from pre-mRNA splicing of four constitutive and four alternative exons. Isoforms are named according to the total number of amino acids in the mature proteins: nine polypeptides of 111, 121, 145, 148, 162, 165, 183, 189 and/or 206 amino acids can be generated (Figure 1). Although nothing is known about the mechanisms regulating the levels of the different VEGF-A isoforms, most VEGF-A producing cells appear to preferentially express VEGF-A121, VEGF-A165 and VEGF-A189. Mice VEGF-A120, 164 and 188 are the murine counterparts of the human VEGF-A121, 165 and 189, respectively (one less amino-acid for each mouse VEGF-A isoform). These splice variants differ by the presence or absence of exons 6a, 6b and 7 (Figure 1). These sequences encode basic residues giving rise to a heparin affinity domain responsible for the differences in the bioavailability and properties of the three main isoforms (26) resulting in distinct spatial distributions of VEGF-A isoforms (Figure 1) (27).

VEGF-A121, which lacks exons 6 and 7, does not bind heparin and is freely released from the cell and is fully diffusible, whereas VEGF-A165 and 189 isoforms are able to bind to heparan sulfate on the cell surface and in the extracellular matrix (28). Each isoform contributes to the formation of a VEGF-A gradient essential for the process of tumour neo-vascularization with soluble isoforms acting at distal sites to promote vascular recruitment, and the extracellular membrane-associated isoforms acting to promote local expansion of capillary beds (29). The fact that an indentical isoform can have distinct activities at different anatomical sites suggests that the microenvironment of different tissues can dictate VEGF-A function (30). To clearly demonstrate the crucial role of alternative splicing in the regulation of VEGF-A activity, different transgenic mice have been generated to express a single VEGF-A isoform.

Mice embryos expressing only VEGF-A120 (*VEGFA^120/120^*) display an impaired post-natal cardiac angiogenesis resulting in severe myocardial ischemia, early post-natal death and impaired lung vascular development (31). Half of the embryos died in the perinatal period due to congenital birth defects and the other half succumbed within 2 weeks after birth, in part due to myocardial ischemia (32). While mice expressing only VEGF-A164 (*VEGFA^164/164^)* are healthy (33), half of the mice expressing VEGF-A188 isoform (*VEGFA^188/188^*) died between embryonic stage E9.5 and E13.5 (33). Taken together, these data suggest that different alternatively spliced isoforms are required for normal development.

Finally, VEGF-A isoforms are functionally redundant in the processes leading to the initial formation of arch arteries (34); they are expressed in distinct spatio-temporal patterns during embryonic development and in adult tissues (35).

Furthermore, exon 8 contains an alternative 3′ splice sites in the nucleotide sequence, which can be used by the cell to generate a distinct family of VEGF-A isoforms with identical length but differing in their C-terminal amino-acid sequences (36). The VEGF-Axxxb isoforms are generated by the use of a distal splice site, 66 nucleotides further along the gene from the proximal splice site (Figure 1). This splicing results in exon 8a excision and produces an mRNA encoding the VEGF-Axxxb family (36). Consequently, the two resultant families of VEGF-A proteins encode either a carboxyl terminus Cys–Asp–Lys–Pro–Arg–Arg for exon 8a or Ser–Leu–Thr–Arg–Lys–Asp for exon 8b. This alternative splicing undoubtedly alters the VEGF-A tertiary structure as the Cys-160, which forms a disulphide bond with Cys-146 in exon 7, is replaced by an acidic residue (Asp) in the VEGF-Axxxb isoforms. The replacement of the two highly charged C terminal arginines in VEGF-A165 by neutral lysine-aspartic acid in VEGF-A165b, and the substitution of a proline by an arginine also contributed to a profound alteration of the structure–function relationship of VEGF-A (37).

**Figure 2.**
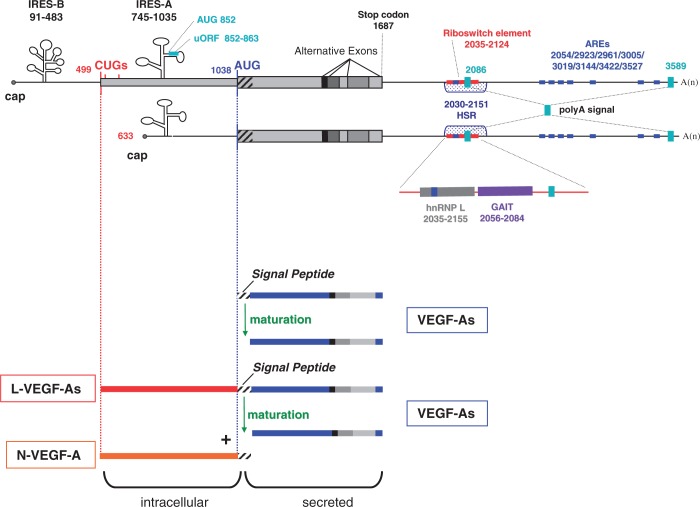
Human VEGF-A mRNAs and their major regulatory elements. mRNAs transcribed from two alternative promoters are schematized. Regulatory elements in the 5′ or 3′ untranslated regions and the coding region are indicated: the position of each regulatory element is numbered according to the VEGF-A189 5′ end mRNA sequence. - Two major alternative initiation codons (AUG and the non-canonic CUG) - IRES-B: Internal Ribosome Entry Site-B driving the expression of the CUG-initiated isoforms - IRES-A: Internal Ribosome Entry Site-A driving the expression of the AUG-initiated forms - uORF: upstream open reading frame, located in the IRES-A - Alternative spliced exons (see also Figure 1 for details) - PolyA signal: two alternative polyadenylation sites - AREs: AU-Rich Elements (consensus sequence AUUUA) - HSR: Hypoxia Stability Region - Riboswitch element: sequence involved in the stress responsive riboswitch driven by exclusive interaction with either hnRNPL or the IFN-γ-activated inhibitor of the GAIT translation complex. Beneath the mRNA, the two protein isoforms, L-VEGF-As and VEGF-As, are represented with their maturation products and localization.

VEGF-A165b was the first xxxb isoform identified by Bates and collaborators (36) and subsequent studies demonstrated the existence of VEGF-A121b, VEGF-A183b, VEGF-A145b (38) and VEGF-A189b (39).

Interestingly, whereas VEGF-Axxx isoforms are pro-angiogenic and are up-regulated in tumours, *in vitro* and *in vivo* studies demonstrated that VEGF-Axxxb isoforms (e.g. VEGF-A165b and VEGF-A121b) are anti-angiogenic and down-regulated in tumours (36,40,41).

Over-expression of VEGF-A165b in tumour cells inhibits the growth of prostate carcinoma, Ewing's sarcoma and renal cell carcinoma in xenografted mouse tumour models (42). Moreover, VEGF-A165b over-expression inhibited tumour cell-mediated migration and proliferation of endothelial cells (42). This anti-angiogenic activity generates receptor binding but impairs signalling events downstream of the receptors (40,43). The VEGF-Axxxb isoforms have been shown to retain both dominant negative and partial receptor agonist activity when co-expressed with VEGF-Axxx isoforms. These properties potentially explained the inhibitory function for receptor binding by competition (44,45).

Recombinant human VEGF-A165b has also been demonstrated to be anti-angiogenic in eye hypoxia-driven angiogenesis (46). It possesses a similar affinity towards the anti-Vascular Endothelial Growth Factor antibody (bevacizumab) indicating that the balance of the switch between anti-angiogenic and pro-angiogenic isoforms can regulate tumour growth rates but can also affect the sensitivity of tumours to bevacizumab by competitive binding (47). Finally, mammary alveolar development during lactation is inhibited by the endogenous VEGF-A165b isoform (48).

More recently, in the lung tissue of a legally aborted female fetus, a novel splicing VEGF-A isoform possessing 20 additional nucleotides from the third intron was described (49). This 20 bases insertion induced a frame shift mutation, introducing a stop codon in the middle of the fourth exon (49). The mechanism involved in this splicing regulation and whether this alteration impact VEGF-A protein structure and/or function are still unknown.

Despite the importance of pre-mRNA VEGF-A alternative splicing, little is known about cellular and molecular events regulating the alternative splicing leading to distinct VEGF-Axxx or VEGF-Axxxb isoforms.

A specific sequence located on the VEGF-A pre-mRNA and involved in alternative splicing of exons has been characterized for exon 6A (50). This exon encloses an exonic splicing silencer sequence (ESS), but regulatory proteins interacting with this sequence are still uncharacterized (50). Studies focusing on VEGF-A splicing have also demonstrated the involvement of different regulatory Serine/Arginine rich proteins (SR proteins). In the endometrial cancer cell line RL95, Elias and coworkers have shown that increased VEGF-A expression under hypoxic conditions is correlated with a shift towards production of the VEGF-A121 isoform. This shift was accompanied by an increase in SR protein expression (ASF/SF2, SRp20 and SRp40 proteins) and phosphorylation (51). In addition, two members of the U2AF65 protein family named CAPERalpha and CAPERbeta were also found to increase VEGF-A121 expression compared with the longer isoforms (52). Nowak and collaborators also demonstrated that ASF/SF2 and SRp40 proteins favour the use of the proximal splice-site in exon 8 resulting in the expression of pro-angiogenic VEGF-A isoforms, whereas SRp55 protein up-regulated the expression of anti-angiogenic VEGF-A isoforms by promoting utilization of the exon 8 distal splice-site (53). Additionally, the transcription factor E2F1 favours the expression of antiangiogenic VEGF-Axxxb isoforms by a mechanism involving the expression of the splicing factor SC35 (54).

Finally, a link between the Wilms’ tumour suppressor gene (WT1) and the regulation of VEGF-A alternative splicing was recently established (55). It was demonstrated that WT1 repressed SRPK1 (Serine/Arginine-rich protein-specific kinase 1) expression by binding its promoter region resulting in a decrease in AFS/SF2 phosphorylation and nuclear localization and consequently leading to anti-angiogenic VEGF-A165b expression. Conversely, mutation of the WT1 tumour suppressor gene results in ASF/SF2 hyperphosphorylation and expression of pro-angiogenic VEGF-A isoforms (55). These data suggest that angiogenesis may be controlled in tumours either lacking functional WT1 or with enhanced SRPK1 expression due to regulation of VEGF-A splicing.

Nevertheless, despite the large number of studies addressing the expression and function of the different VEGF-A isoforms, our understanding of the mechanisms regulating alternative splicing is still in its early days.

### mRNA stability

Generally variations in the mRNA level are due to altered mRNA stability in response to environmental changes such as nutriment levels, cytokines, hormones or stress (56,57). AU rich elements (AREs) are the most described cis-sequences involved in the regulation of mRNA stability (58,59). These elements, identified initially from mRNA sequences having short half-lives, are crucial for the mRNA degradation process. ARE elements are generally composed of different nonameric or pentameric consensus or U-rich sequences (58,59).

VEGF-A mRNA is highly labile under normal oxygen and nutrient conditions with a half-life of 15–40 min *in vitro* (60–63). Under normoxic conditions, the destabilizing potential of the 5′UTR, the coding region and the 3′UTR mouse VEGF-A was assessed by determining the relative expression level of the mRNA expressed constitutively. Rapid degradation of VEGF-A mRNA under normoxic conditions is linked to the combined action of these independent destabilizing elements. Moreover, under hypoxia, stabilization of the mouse VEGF-A mRNA requires the cooperation of elements in all three regions of the mRNA (60). The stability of human VEGF-A mRNA is mainly mediated through the 3′UTR which possesses several AU rich elements (Figure 2) targeted by ARE-binding proteins such as AUF1 and tristetraprolin (TTP), leading to destabilization of VEGF-A mRNAs in various mammalian cell types (64–68). AUF1 assembles other factors necessary to recruit the mRNA degradation machinery including translation initiation factor eIF4G, heat-shock cognate protein hsc70, lactate dehydrogenase, poly(A)-binding protein, etc. Additionally, AUF1 is subjected to many post-translational modifications such as phosphorylation, glycosylation, methylation and ubiquitination and is thus regulated by many signalling pathways [for review, see (69)].

Interestingly, Fellows and collaborators showed recently that AUF1 regulates the expression of VEGF-A in mouse macrophage-like RAW-264.7 cells through its C-terminus domain containing a region with three arginine–glycine–glycine (RGG) motifs (70). Similarly, TIS11/Tristetraprolin (TTP), another AU-rich element binding protein regulating VEGF-A mRNA half-life, is also subjected to regulatory pathways as it has recently been shown that Casein kinase 2 indirectly regulates the mRNA-destabilizing activity of Tristetraprolin through p38 MAPK (71).

Conversely, ARE elements contributed to mRNA stabilization through interactions with the ELAV protein family, which includes Hel-N1, HuC, HuD and HuR protein (72–75). ELAV/Hu proteins regulate the stability of ARE containing mRNA, from splicing to translation, by generating multimeric ‘ribonucleosomes’ due to their properties of nucleation and cooperative binding on target mRNAs. A model has been proposed whereby ELAV/Hu proteins have the ability to displace miRNP targets and thus suppress their destabilizing properties (76). In a recent report Chang and collaborators seem to confirm this model for the mouse VEGF-A mRNA as HuR and miR-200b binding sites overlap. The ability of miR-200b to suppress VEGF-A expression was competitively antagonized by HuR at the 3′UTR, thus constituting an RNA regulon that is important for VEGF-A expression (77). Moreover, HSP70 was found to bind and stabilize VEGF-A mRNAs through a mechanism unaffected by an inhibitor of its chaperone function (78).

Interestingly, several investigations suggest that poly(A)-binding protein, a stabilizing factor for polyadenylated mRNAs, has destabilizing effects on VEGF-A mRNA (66,79).

Hypoxia-induced mRNA stability has been shown to be a mechanism that can increase VEGF-A expression in tumours independently of *HIF1A-*induced transcription (80). AU-rich elements within the VEGF-A 3′-UTR also confer hypoxia-dependent mRNA stability (81–83). RNA-binding proteins interacting with these 3′-UTR elements include the CSD/PTB complex (Cold Shock Domain/Polypyrimidine Tract Binding Protein) (84), HuR (85), hnRNP L (86), PAIP2 [Poly(A)-Binding Interacting Protein 2] (87,88), TIS11b/TTP (89) and finally, the double strand RNA-binding protein DRBP76/NF90 (90). PAIP2 interacts directly with HuR recruited in distinct mRNA regions, suggesting that interaction between these proteins could influence the overall RNA structure and render the RNA inaccessible to endonucleases (87). The DRBP76/NF90 isoform has been shown to facilitate VEGF-A expression by promoting VEGF-A mRNA loading onto polysomes and translation under hypoxic conditions (90).

Moreover, MDM2, a protein translocated from the nucleus to the cytoplasm under hypoxic conditions, is able to bind AU-rich sequences within the 3′ UTR of VEGF-A mRNA and to increase mRNA stability and translation during hypoxia (91).

Finally, it has been postulated that effective VEGF-A mRNA export from the nucleus and loading onto active polysomes under hypoxia can be increased by extra-nuclear shuttling of mRNA-binding proteins such as HuR, hnRNP L and hnRNP A1, which regulate VEGF-A mRNA stability (90).

### Alternative polyadenylation

The complexity of VEGF-A regulation results in part from the use of alternative polyadenylation sites within the *VEGF-A* gene. Initially, a human mRNA with a 360 nucleotides long 3′UTR was cloned, (92) but longer cDNAs carrying a 2 kbp long 3′UTR have also been characterized (93). Subsequently, sequencing of the full-length cDNA revealed the presence of two major polyadenylation sites: the consensus site AAUAAA and the non-canonical site AUUAAA, located 399 and 1902 nucleotides after the stop codon (Figure 2) (81,94). Despite the presence of putative polyadenylation sites, the knowledge of poly(A) signal utilization in the 3′ end region of the pre-mRNA is limited to one study on the mouse VEGF-A (94). Dibbens and collaborators showed that the majority of the mouse VEGF-A transcripts are processed at the distal poladenylation site (resulting in mRNAs containing the longer form of the 3′UTR), with the same site being used under both hypoxic and normoxic growth conditions (94).

In conclusion, our understanding of the use of poly(A) signals is very limited even though mRNA 3′UTRs are known to play a pivotal role in the post-transcriptional regulation of gene expression (95) i.e. mRNA stability and subcellular localization, alternative splicing and translation initiation (discussed below).

### Translational regulation

Translation in eukaryotes is a complex multi-step process. Like most complex biochemical reactions, it is subject to strict regulatory controls, and extremely sensitive to both intracellular and extracellular environments. Transcript-selective translational control is generally mediated by interactions of RNA-binding proteins with structural elements within non-coding regions. Besides protein–RNA interactions, RNA–RNA interactions also regulate VEGF-A expression (riboswitches, miRNA).

#### IRES elements and alternative initiation at non-AUG codons

Translation of mRNA within eukaryotic cells is mainly dependent on the m7G cap, a unique structure located at the 5' terminus of the mRNA, but a number of cellular mRNAs can be translated by a cap-independent mechanism. These mRNAs enclose an IRES within their 5′ untranslated region. IRESs have been mostly reported in mRNAs that have long 5′UTRs with a high GC content and an extensive predicted secondary structure. These structural elements are specifically required to maintain and/or activate the expression of specific proteins during cell stress situations when cap-dependent translation is compromised (96). Until now >70 viruses and several eukaryotic mRNAs are reported to include IRESs (97). The VEGF-A mRNA coding region is flanked by a 1038-nucleotide-long GC rich 5′UTR containing three in-frame alternative CUG start codons and two internal ribosome entry sites, which enable alternative initiation of translation (25,98,99). The first CUG start codon is located 539 nucleotides upstream of the classical AUG initiation codon (100–103). The first identified IRES (IRES-A) is within the 300 nucleotides upstream from the AUG start codon and IRES-B is located in the first half of the 5′ UTR up to 16 nucleotides upstream from the first CUG codon (Figure 2).

IRESs control the translation at the AUG initiation codon and the two alternatives CUG start codons, as demonstrated *in vitro* (25,98) and *in vivo* (104). VEGF-A IRESs have low activity in embryos and adult tissues, but allow efficient translation at early time points in ischaemic muscle, a stress that inhibits cap-dependent translation. This translation revealed the efficiency of the VEGF-A IRESs in response to a local environmental stress *in vivo* such as hypoxia (104). Recently, IRES trans-acting factors controlling translation of VEGF-A mRNA were identified. Using a high-throughput screening approach that combined siRNA treatment and transfection of a VEGF-A IRES reporter mRNA, Casanova and collaborators identified the kinase MAPK3 as a novel positive regulator. In addition, they have validated its regulatory effect on endogenous VEGF-A (105). The DEAD-box RNA helicase 6 (DDX6) has also been identified as an IRES trans-acting factor in the MCF-7 cell line. Recombinant DDX6 inhibits VEGF-A IRES mediated translation under normoxic conditions in MCF-7 extracts. Under hypoxia the decrease in the DDX6 level leads to the induction of VEGF-A expression (106).

Finally, initiation of alternative translation at the most upstream CUG codon leads to the synthesis of L-VEGF-A (Figure 2), a precursor cleaved at the peptide signal sequence to generate short secreted VEGF-A isoforms known to have biological effects, as well as the intracellular N-VEGF-A, a 23-kDa NH2-specific fragment consisting of 206 amino acids (Figure 2) (100,102,107).

The reciprocal roles of L-VEGF-A and N-VEGF-A are still unclear. L-VEGF-A may serve as a reservoir to generate shorter isoforms as described by Chiarini et al. who showed that an increase in intracellular L-VEGF-A189 and L-VEGF-A165 protein isoforms was linked to a reduction in secreted VEGF-A isoforms (108).

High conservation of the N-VEGF-A amino acid sequences between mammals suggests that N-VEGF-A could have a specific function. Interestingly, a single nucleotide polymorphism in the human VEGF-A gene (-634 C-G) results in IRES-B dysfunction that leads to a 17% reduction in initiation at the CUG codon and thus a decrease in L-VEGF-A expression (109). This polymorphism was associated with (i) an increased risk in motor neuron degeneration in amyotrophic lateral sclerosis (ALS) (109), (ii) the development of diabetic macular oedema correlated with macular retinal thickness in type 2 diabetes (110), (iii) a higher breast cancer aggressiveness (111) and (iv) an increased risk of gastric (112) and prostate cancer (113). These studies provided evidence of a crucial role for IRES function and consequently the significance of the level of L-VEGF-A isoforms.

Finally, differential use of translation initiation at alternative initiation codons is also reported (114). Translation initiation at both AUG and CUG codons is dependent on the exon content of the coding region. While initiation at the CUG codon is equally efficient regardless of which splice variant is expressed, use of the AUG codon depends on the exon content of the VEGF-A mRNA (114). Indeed, the three major VEGF-A splice variants have distinct properties in terms of the selection of the translation start codon. VEGF-A121 is exclusively expressed through initiation at the CUG codon, and thus is a maturation product of the high molecular weight L-VEGF-A121. In contrast, the VEGF-A165 and 189 splice variants are efficiently expressed through initiation events using both start codons. This differential expression pattern is exerted through the modulation of IRES-A activity by the alternatively spliced coding sequences (114). It has been postulated that long-range interactions in the VEGF-A mRNA could regulate IRES-A activity and thus control start codon use, but the exact molecular mechanism of this control is still unknown.

#### Upstream open reading frame

Upstream open reading frames (uORFs), generally short and juxtaposed to the actual ORF, are found in numerous eukaryote mRNAs and are known to regulate protein translation under normal and stress conditions (115,116). Translation of several proto-oncogenes, cytokines and many other factors, such as human 5-HT3A (5-hydroxytryptamine receptor 3A), TPO, BACE1 [β-site APP (Amyloid Precursor Protein) cleavage enzyme 1] or huntingtin protein (protein associated with Huntington's disease) are in part tightly controlled by the presence of uORFs in their 5′UTRs (117). Interestingly, analysis of the human VEGF-A 5′UTR revealed the presence of a unique short uORF (Figure 2). This uORF is highly conserved between species and begins at AUG 852, 186 nucleotides upstream of the main AUG. The uORF is located within the IRES-A sequence and translated through a cap-independent mechanism, and is an essential regulator controlling isoform expression (118). Generally, uORFs act as a constitutive barrier to the scanning ribosome and reduce the number of ribosomes that gain access to the main AUG codon. VEGF-A uORF acts differently given that mutation of the AUG 852 (Figure 2) increased translation of the VEGF-A121 isoform, but had no effect on the expression of VEGF-A165 or 189. Our current inability to predict alteration of uORF function means that this uORF (potentially constitutive inhibitor) may be regulated either by specific sequences, trans-acting factors or conditions that have not yet been identified. Thus, the VEGF-A uORF seems to acts as a cis-regulatory element involved in the CUG shut-off of VEGF-A121 mRNA (118) and by this mechanism controls expression of VEGF-A isoforms. Taken together, these data demonstrated that this uORF affects VEGF-A expression and is an additional element contributing to its fine-tuning.

#### miRNA-mediated regulation of VEGF-A

The regulation of post-transcriptional gene expression by microRNAs (miRNA) has emerged as a widespread mechanism. miRNAs belong to the group of non-coding RNAs, that are small in size (20–25 nucleotides), and are generated from local hairpin structures by two RNA endonucleases, namely Drosha and Dicer (119). VEGF-A mRNA as target of miRNAs was first proposed in 2004 by Yang and co-workers who demonstrated that invalidation of the dicer gene in mice caused retarded development, defective angiogenesis and the death of embryos between days 12.5 and 14.5 of gestation. This phenotype was correlated with VEGF-A over-expression indicating that defective angiogenesis was most likely due to deficient VEGF-A mRNA processing (120).

**Figure 3.**
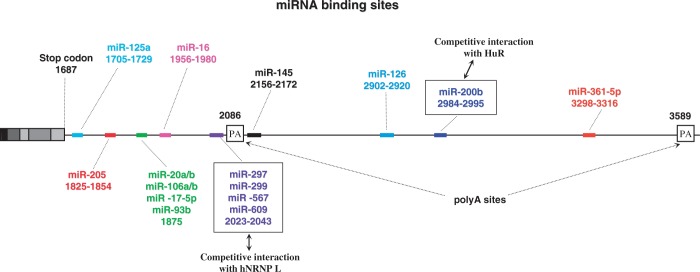
miRNA-targeting sites in the human VEGF-A mRNA 3′UTR. Position of each miRNA target site is represented and numbered according to the VEGF-A189 5′ end mRNA sequence.

Numerous studies have been devoted to identify miRNAs targeting VEGF-A mRNA; their binding sites within the 3′UTR region are summarized in Figure 3.

The miR-20 a/b, miR-106 a/b, miR-17-5p, miR-16 and miR-15b were the first to be reported to bind to the VEGF-A 3′untranslated region (121,122). Interestingly, it was demonstrated that miR-15b, miR-16, miR-20a and miR-20b co-regulated other angiogenic factors (121). Down-regulation of miR-126 in lung cancer cell lines, miR-200b in the diabetic retina, miR-93b under hyperglycemic conditions or miR-205 in glioma cell lines increased VEGF-A expression (123–126). Additionally, miR-361-5p levels are inversely correlated with VEGF-A expression in human cutaneous squamous cell carcinoma (127), miR-125a inhibits the proliferation and metastasis of hepatocellular carcinoma by targeting VEGF-A (128), and miR-145 inhibits tumour angiogenesis, cell growth, invasion and tumour growth through the post-transcriptional regulation of VEGF-A (129,130). Finally, miR-16 targets a responsive element located 270 nucleotides downstream of the translation stop codon (131), and hence affects angiogenesis in multiple myeloma (132).

All these studies overshadow the cap-independent translation mechanism and describe VEGF-A inhibition by miRNAs. Interestingly, Karaa and coworkers demonstrated that the two IRESs activities are differently affected by miR-16. While IRES-A is insensitive to miR-16 inhibition, IRES-B (controlling expression of the diffusible VEGF-A121 isoform) can be negatively regulated by miR-16 (131). This is the first example of the inhibition of a specific isoform translation by an miRNA.

In addition, Jafarifar and collaborators have demonstrated that at least four additional miRNAs, (miR-297, miR-299, miR-567 and miR-609) target the CA-Rich Element (CARE) of the human VEGF-A 3′UTR and provide evidence of a robust inhibition of protein synthesis under normoxic conditions (133). Remarkably, these authors demonstrated that under hypoxia, hnRNP L is delocalized from the nucleus to the cytoplasm, binds to the CARE sequence and then presumably blocks the accessibility for the miRISC (RNA-Induced Silencing Complex with incorporated miRNA) both targeting the same element (133). This mechanism underlines the key role of hnRNP L during hypoxia that is also illustrated by its ability to promote VEGF-A mRNA stability (see above) and its role in the VEGF-A mRNA riboswitch (see below).

A recent study has reported a similar competition mechanism between an RNA-binding protein involved in VEGF-A mRNA stability and an miRNA recognizing the same binding site (Figure 3). The authors have identified the antagonist pair HuR/miR-200b that controls VEGF-A expression in bone marrow-derived macrophages (77). Interestingly, this mechanism is evolutionarily conserved, at least between mouse and Zebra fish.

#### Riboswitch

Messenger RNAs can respond to changes in their environment by altering their folding structure and their rate of translation. These RNA switches, also known as riboswitches, typically established in the untranslated regions are able to bind small ligands or proteins and regulate gene expression in bacteria, fungi and plants.

Like riboswitches, the VEGF-A 3′UTR adopts a binary conformational change in response to environmental signals. This atypical riboswitch is metabolite independent and the conformational change is controlled by differential protein binding. In myeloid cells, the interferon-γ-activated inhibitor of translation (GAIT) complex and hnRNPL protein alter the production of VEGF-A during oxidative stress by repressing or activating the translation respectively (134). The proximity of the hnRNPL protein and GAIT complex binding sites (Figure 2) suggests that interactions are mutually exclusive. This process is regulated by stimulus-dependent proteasomal degradation of hnRNPL. During hypoxia, hnRNPL intracellular accumulation promotes one of the two conformations and thus suppresses the GAIT-mediated translation silencing of VEGF-A mRNA (134,135). Finally, given that the riboswitch sequence encompasses the first polyadenylation site, one can postulate that RNA conformational changes may influence the use of one polyadenylation site and consequently affect the presence of regulatory elements within the 3′UTR (AU-rich elements and miR-binding sites).

These studies highlight the continuing expansion of our knowledge of the scope of ribo-regulation in eukaryotes.

#### RNA G-quadruplex structure

G-rich regions can fold up to form secondary structures organized in stacks of planar layers of guanine tetrad (or quartet) units termed G-quadruplexes. G-quadruplexes are emerging regulatory element present in different mRNA regions (coding and non-coding) and involved in different steps of RNA metabolism, including mRNA translational regulation (136). G-quadruplex forming sequences located in 5′UTRs have been mainly described as translation inhibitors of cap-dependent translation [e.g. in Zic-1, ERalpha (ESR1), NRAS or MT3-MMP mRNA] probably through the recruitment of stabilizing proteins preventing ribosome scanning (137). Recently, a 17-nucleotide element located within the VEGF-A IRES-A (nucleotides 774–790) has been shown to fold in a two G-quartet quadruplex structure. Mutations disrupting the intramolecular G-quadruplex structure inactivated IRES-A function, suggesting the requirement of this structure to maintain IRES-A activity (138). This finding is quite surprising because the majority of the naturally occurring G-quadruplexes displaying translationally regulatory properties are at least three-G-quartet quadruplex structures while artificially engineered two-G-quartet quadruplexes displayed only a moderate stability (137).

Because IRESs ensure translation activation of mRNA during stress, an important unsolved issue is to establish whether this structure contribute to IRES-A activation under stress conditions known to increase VEGF-A expression, such as hypoxia, endoplasmic reticulum stress or ischemia.

### A new class of non-coding RNA?

It was recently shown that a non-coding regulatory RNA, mapped in the 5' UTR of VEGF-A mRNA, plays a function in tumour development by affecting the expression of other genes independently of VEGF-A translation (139). It was postulated that VEGF-A transcripts might have cancer-related functions, as down-regulation of VEGF-A decreased resistance to chemotherapeutic 5-fluoro-uracil-induced apoptosis and that recombinant VEGF-A did not completely reverse this phenotype. Accordingly, the expression of the VEGF-A 5′UTR, without its open reading frame, induces an increase in colony-forming activity *in vitro* together with tumour formation in a mouse xenograft model (139). These results indicate that VEGF-A mRNA itself may promote malignancy of tumour cells.

Undoubtedly, non-coding RNAs have now been recognized to play critical roles in tumourigenesis, but there are still few examples of trans-acting functions of mRNA untranslated regions associated with tumourigenic properties. Some mRNA untranslated regions, including the 3′UTR of α-tropomyosin mRNA (140), ribonucleotide reductase mRNA (141), prohibitin mRNA (142) and the 5′UTR of c-myc mRNA (143), possess tumour suppressor functions. Conversely, the VEGF-A 5′UTR is the first example harbouring tumour-promoting activities. The current challenge will be to characterize the molecular mechanism of this process and to investigate how common these findings are.

## DISCUSSION AND CONCLUSION

In the past decades, RNA was first recognized as a passive intermediate of genetic information between DNA and proteins. Over the past 20 years numerous studies have contributed to a better characterization of the importance of post-transcriptional regulation in gene expression.

The critical biological functions of VEGF-A during embryogenesis and in the adult result in tight control of its expression. Indeed, the presence of two promoters, two polyadenylation signals and 14 alternative splicing events enable 56 potential mRNAs to be expressed. At the post-transcriptional level, VEGF-A regulation is a model given that most sophisticated regulatory mechanisms are used. This complex post-transcriptional regulation includes alternative polyadenylation, alternative splicing, regulation of mRNA stability, IRES-dependent translation, uORF and isoform expression, miRNA-mediated regulation, riboswitching and finally G-quadruplex structure and IRES activity. These regulatory mechanisms are linked to the presence of the largest number of regulatory elements (HSR, ARE sequences, IRES, hnRNPL or GAIT-binding sites) in VEGF-A mRNA. The complexity is amplified by cross-talked regulations such as differential IRES sensitivity to miRNA inhibition leading to the control of specific isoforms expression.

Moreover, the use of alternative initiation codons enables expression of at least 29 putative pre-proteins (14 AUG initiated forms, 14 CUG initiated forms or L-VEGF-A and the amino terminal extension N-VEGF-A) exhibiting distinct biological functions. This post-transcriptional regulatory network allows fine-tuning of the expression of VEGF-A isoforms during embryogenesis or under physiological conditions in the adult.

For all these reasons, VEGF-A is a paradigm for intricate regulation of gene expression at the post-transcriptional level and is, to our knowledge, the mRNA containing the largest variety of regulatory elements.

However, the multiplicity of mechanisms regulating VEGF-A mRNA is certainly not an exception. Indeed, alternative splicing was shown in >90% of genes (144) and a recent study showed that ∼79% of the transcripts have multiple polyadenylation sites (145). Translational control, another mechanism increasing the complexity level of an organism by alternative isoform expression, is less frequent.

To our knowledge, there are ∼100 human mRNAs initiating translation from alternative start codons and at least 115 eukaryotic cellular mRNAs reported to contain IRESs even if there is still controversy regarding the validity of some IRESs claimed as being cellular IRESs (146,147).

These translational mechanisms will certainly be found to be more widespread due to the development of new approaches and technologies (148–150). Future studies will probably reveal that combinations of complex regulations affect numerous mRNAs subjected to fine-tuning controls. Thus, the complexity and multiplicity of regulatory mechanisms involved in VEGF-A expression places VEGF-A as a model to study the regulation of gene expression.
